# Supplementary material for: Flexible circuit mechanisms for context-dependent song sequencing
Source: Nature. 2023 Oct 11;622(7984):794–801. doi: 10.1038/s41586-023-06632-1 (PMC10600009; doi:10.1038/s41586-023-06632-1)
Supplement: Supplementary file 1 — This file contains Supplementary Tables 1–4, listing reagents for experiments, the genotypes of all of the fly strains used, and parameters for computational modeling, and Supplementary Methods, providing details on experiments and analysis related to Extended Data Figures 1–8. [file 41586_2023_6632_MOESM1_ESM.pdf]

nature portfolio

https://doi.org/10.1038/s41586-023-06632-1

Supplementary information

Flexible circuit mechanisms for context-dependent song sequencing

In the format provided by the authors and unedited

Supplementary information

Frederic A. Roemschied[1,2], Diego A. Pacheco[1,3], Max J. Aragon[1], Elise C. Ireland[1],
Xinping Li[1], Kyle Thieringer[1], Rich Pang[1], Mala Murthy[1,*]

[1] Princeton Neuroscience Institute, Princeton University, Princeton, NJ, 08544, USA
[2] Present address: European Neuroscience Institute, Grisebachstr. 5, 37077 Göttingen, Germany
[3] Present address: Harvard Medical School, Warren Alpert Building 320, 200 Longwood Ave, Boston, MA 02115, USA
[*] Corresponding author: mmurthy@princeton.edu

Supplementary Tables

Supplementary Table S1: Key resources table

Reagent type (species) or resource	Designation	Source or reference	Identifiers	Additional Information
Genetic reagent (*D. mel*)	NM91	[1]		Obtained from Peter Andolfatto
Genetic reagent (*D. mel*)	w[1118]; PBac{w[+mC]=WH} Ih[f01485]	Exelixis at Harvard Medical School	FlyBase: FBti0049996	Obtained from María Fernanda Ceriani
Genetic reagent (*D. mel*)	w[1118]; PBac{w[+mC]=WH} Ih[f03355]	Exelixis at Harvard Medical School	FlyBase: FBti0051182	Obtained from María Fernanda Ceriani
Genetic reagent (*D. mel*)	20xUAS-IVS-CsChrimson.mVenus (X) (attP18)		BDSC: 55134	Obtained from Vivek Jayaraman and Gerry Rubin
Genetic reagent (*D. mel*)	VT040556-p65ADZp (attp40)	[2]		Obtained from David Stern
Genetic reagent (*D. mel*)	VT040347-ZpGDBD (attp2)	[2]		Obtained from David Stern
Genetic reagent (*D. mel*)	GMR15A01-AD (attp40)	[3]		Obtained from David Anderson
Genetic reagent (*D. mel*)	GMR71G01-DBD (attp2)	[3]		Obtained from David Anderson
Genetic reagent (*D. mel*)	GMR42B01-Gal4 (attP2)	[4]		Obtained from Bruce Baker
Genetic reagent (*D. mel*)	dsx-LexA	[4]		Obtained from Bruce Baker
Genetic reagent (*D. mel*)	8xLexAop2-flp	[4]	BDSC 55819-20	Obtained from BDSC
Genetic reagent (*D. mel*)	GMR13H01.LexA::p65 (attP40)	[5]		Obtained from Troy Shirangi
Genetic reagent (*D. mel*)	dsx-Gal4(Δ2)	[6]		Obtained from Troy Shirangi
Genetic reagent (*D. mel*)	+/+; fruFLP/VT040556-Gal4	[7]		fruFLP and VT040556-Gal4 were provided by Barry Dickson
Genetic reagent (*D. mel*)	GMR53G02-AD (attP40)/UAS-Kir2.1	[7]		GMR53G02-AD (attP40) obtained from BDSC and contributed by Gerry Rubin
Genetic reagent (*D. mel*)	GMR53G02-AD (attP40)/UAS-TNT	[7]		
Genetic reagent (*D. mel*)	UAS>stop>Kir2.1/+; fruFLP/VT040556-Gal4	[7]		UAS>stop>Kir2.1 was provided by Troy Shirangi
Genetic reagent (*D. mel*)	UAS>stop>TNT/+; fruFLP/VT040556-Gal4	[7]		
Genetic reagent (*D. mel*)	norpA[36]-20xUAS-CsChrimson.mVenus (attP18);VT040556-AD (attP40)/GMR15A01-AD (attP40);VT043047-DBD (attP2)/GMR71G01-DBD (attP2)			
Genetic reagent (*D. mel*)	;;13xLexAop-IVS-GCAMP6s (attp1)		BDSC 44273	
Genetic reagent (*D. mel*)	;LexAop-tdTomato.Myr, su(Hw) (attp5), BRP>STOP>V5-2A-LexA-VP16 [VK00018]/Cyo; TM2/TM6B, tb		BDSC 56142	
Genetic reagent (*D. mel*)	w[*] NorpA[36], 20xUAS-IVS-CsChrimson.mVenus (attp18); GMR71G01-AD (attp40)/13xLexAop-IVS-GCAMP6s; GAD1-LexA/GMR15A01-DBD (attp2)			
Genetic reagent (*D. mel*)	y[1] sc[*] v[1] sev[21]; Py[+t7.7] v[+t1.8]=TRiP.HMC03319 (attp2)/TM3, Sb[1] Ser[1]		BDSC: 51765	obtained from BDSC
Genetic reagent (*D. mel*)	y[1] sc[*] v[1] sev[21]; Py[+t7.7] v[+t1.8]=TRiP.HMC03643 (attp40)		BDSC: 52903	obtained from BDSC
Genetic reagent (*D. mel*)	y[1] v[1]; Py[+t7.7]=CaryP (attP2)		BDSC: 36303	obtained from BDSC
Genetic reagent (*D. mel*)	w[*]; Py[+t*] w[+mC]=UAS(FRT.stop)Ctet\tetXVIE-19A/CyO		BDSC: 67690	obtained from BDSC
Genetic reagent (*D. mel*)	w[1118]; Py[+t7.7] w[+mC]=8XLexAop2-FLPL (attp40)		BDSC: 55820	obtained from BDSC
Chemical compound, drug	All-trans retinal	Sigma-Aldrich	#R2500	

Supplementary Table S2: List of strains used in manuscript

Figure panel	Genotype	Additional Information
Fig. 1; Extended Data Fig. 1b,c,e,f,g,h,i; Extended Data Fig. 5m,n; Extended Data Fig. 7k	NM91	D. melanogaster NM91 males (provided by Peter Andolfatto) courting NM91 wild type females.
Fig. 2b-g; Fig. 4e,f,j-o; Extended Data Fig. 2a,c,e,g,i; Extended Data Fig. 5d,p-s; Extended Data Fig. 7d-f	UAS-CsChrimson/+; VT040556-AD (attp40)/+; VT040347-DBD (attp2)/+	Express CsChrimson in pIP10 neurons; VT040556-AD (attp40) and VT040347-DBD (attp2) [2] provided by David Stern
Extended Data Fig. 8a-c	norpA[36]-20xUAS-CsChrimson.mVenus (attp18)/+; VT040556-AD (attp40)/+; VT040347-DBD (attp2)/+	Express CsChrimson in pIP10 neurons of norpA-blind flies; VT040556-AD (attp40) and VT040347-DBD (attp2) [2] provided by David Stern
Extended Data Fig. 7g-h	UAS>stop>Kir2.1/+; fruFLP/VT040556-Gal4	Express kir2.1 in pIP10 neurons [7]
Extended Data Fig. 7g-h	GMR53G02-AD (attp40)/UAS-Kir2.1	control kir2.1
Extended Data Fig. 7g,h	+/+; fruFLP/VT040556-Gal4	pIP10 control
Extended Data Fig. 7g-h	UAS>stop>TNT/+; fruFLP/VT040556-Gal4	Express TNT in pIP10 neurons
Extended Data Fig. 7g-h	GMR53G02-AD (attP40)/UAS-TNT	control TNT
Fig. 2h-l ; Extended Data Fig. 2b,d,f,h,j; Extended Data Fig. 5t,u	+/+; GMR13H01-LexA/20xUAS>stop>CsChrimson.mVenus (attp40); Dsx-Gal4/8xLexAop-FLP	Express CsChrimson in TN1 neurons; GMR13H01.LexA::p65 (attp40) and dsx-Gal4(Δ2) [5] provided by Troy Shirangi
Fig. 4b,e,f; Extended Data Fig. 4a-b; Extended Data Fig. 5c,d	UAS > stop > CsChrimson.mVenus (attp14)/8XLexAop2-flp; dsx-LexA, 8xLexAop2-flp/GMR42B01-Gal4	Express CsChrimson in Dsx+ pC2 neurons [4]
Fig. 4d-f; Extended Data Fig. 4; Extended Data Fig. 5e ; Extended Data Fig. 7i	norpA[36]-20xUAS-CsChrimson.mVenus (attp18);VT040556-AD (attp40)/GMR15A01-AD (attp40);VT043047-DBD (attp2)/GMR71G01-DBD (attp2)	Express CsChrimson in pIP10 and P1a neurons
Fig. 4a,e,f; Extended Data Fig. 5a,b; Extended Data Fig. 7i	UAS-CsChrimson(attp18)/+; GMR15A01-AD (attp40)/+; GMR71G01-DBD (attp2)/+	Express CsChrimson in P1a neurons; GMR71G01-DBD (attp2) and GMR15A01-AD (attp40) [3] kindly provided by David Anderson.
Fig. 3b-e	w[*] NorpA[36], 20xUAS-CsChrimson-mVenus (attp18); VT040556-AD(attp40)/ LexAop-tdTomato.Myr, su(Hw) (attp5), BRP>STOP>V5-2A-LexA-VP16 [VK00018]; VT400347-DBD (attp2)/Dsx-FLP, 13xLexAop-IVS-GCAMP6s (attp1)	Express mVenus-tagged CsChrimson in pIP10 neurons and GCaMP6s in Dsx+ neurons of norpA-blind males;
Extended Data Fig. 3d-f	w[1118]; PBac{w[+mC]=WH} Ih[f01485]	D. melanogaster Ih mutant males (provided by María Fernanda Ceriani) courting NM91 wild type females
Extended Data Fig. 3c-f	w[1118]; PBac{w[+mC]=WH} Ih[f03355]	D. melanogaster Ih mutant males (provided by María Fernanda Ceriani) courting NM91 wild type females
Extended Data Fig. 3g-i	+/+; GMR13H01-LexA / tub>Gal80>; Dsx-Gal4, LexAop-FLP / UAS-Ih-RNAi	RNAi-knockdown of Ih in TN1 neurons of males courting NM91 wild-type females
Extended Data Fig. 3g-i	+/+; GMR13H01-LexA / UAS-Rdl-RNAi; Dsx-Gal4, LexAop-FLP / tub>Gal80>	RNAi-knockdown of Rdl in TN1 neurons of males courting NM91 wild-type females
Extended Data Fig. 3h-i	+/+; GMR13H01-LexA / tub>Gal80>; Dsx-Gal4, LexAop-FLP / +'(attp2)	driver control for I_h and Rdl knockdown experiments, where $+'$ is the genetic background control for TRiP UAS-RNAi lines
Fig. 4g-i	+/+ ; UAS>stop>TNT/LexAop-FLP ; Dsx-LexA/GMR42B01-Gal4	Express TNT in Dsx+ pC2 neurons of males courting NM91 wild-type females.
Fig. 4g-i	+/+ ; +/LexAop-FLP ; Dsx-LexA/GMR42B01-Gal4	Background control genotype for the pC2 silencing experiments, with males courting NM91 wild-type females
Extended Data Fig. 8	norpA[36]-20xUAS-CsChrimson.mVenus (attP18);VT040556-AD (attp40)/+;VT043047-DBD (attp2)/+	Express mVenus-tagged CsChrimson in pIP10 neurons of norpA-blind males
Extended Data Fig. 5i-k	w[*] NorpA[36], 20xUAS-IVS-CsChrimson.mVenus (attp18); GMR71G01-AD (attp40)/13xLexAop-IVS-GCAMP6s; Gad1-LexA/GMR15A01-DBD (attp2)	Express mVenus-tagged CsChrimson in P1a neurons, and GCaMP6s in Gad1+ neurons of norpA-blind males

Supplementary Table S3: Izhikevich neuron parameters

Neuron	a	b	c	d	Additional Information
pC2,inh	0.02/ms	0.2/ms	-65mV	6V/s	'tonic spiking' neuron
p,s	0.03/ms	0.25/ms	-60mV	4V/s	'rebound spiking' neuron

Supplementary Table S4: Circuit model and genetic algorithm parameters

Parameter	Value/Range	Additional Information
τ_i	10ms	inhibitory time const.
τ_e	2ms	excitatory time const.
τ_m	5ms	membrane time const.
vth	30mV	spiking threshold
v0	-87mV	initial membrane potential
α	1	input nonlinearity
β	0.5	input nonlinearity
x_0	2	input nonlinearity
dt	0.1ms	Euler integration time step
I_e	$[2, 25]$	Range used for optimization
w_i	$[-150, -5]$	Range used for optimization
w_e	$[5, 150]$	Range used for optimization
I_{tonic}	$[3, 15]$	Range used for optimization
number of iterations	25	
population size	30	
mutation probability	0.1	
elite ratio	0.01	
crossover probability	0.5	
crossover type	uniform	

Supplementary Methods

Fast online sine segmentation

To facilitate closed-loop optogenetic activation triggered on male sine song production, we used a custom-made fast online sine song segmenter that was based on two convolutional neural networks (CNNs). Briefly, at every time point during a recording, 384 samples (38.4ms) of sound history (384x16 samples for 16 microphone channels) served as input to the online segmenter. First, to reduce data dimensionality, the channel with the maximum mean power was selected for further processing and normalized by dividing by its 2-norm. We then applied a 512-point discrete Fourier transformation (DFT) to the normalized 1-dimensional signal and kept the 51 DFT amplitudes for frequencies below 1000 Hz for further processing. The normalized 384-sample time-domain waveform and the 51-sample frequency-domain amplitudes were fed into a classifier model that combined two CNNs. The output of the model was a sigmoidal activation value associated with the probability that the input data contained sine song. We used a threshold of 0.995 on this activation value to create a binary output value that controlled the optogenetic stimulus LEDs during closed-loop neural activation (turning the stimulus on for $p(\text{sine}) \geq 0.995$ and off for $p(\text{sine}) < 0.995$). The CNNs were implemented using Keras [8] with Theano backend [9]. The model was trained on output of the offline song segmenter for $n = 19$ recordings of wild-type (NM91) male-female pairs not used otherwise in the present study. During these recordings, optogenetic stimulus LEDs were randomly turned on and off to generate a realistic noise background (as during closed-loop neural activation). Segmented song split up into 384x16-sample chunks (window size x number of microphones) and each chunk was labeled as 'sine' or 'no sine' to provide ground truth data for training. The recordings were divided into 11, 4, and 4 recordings for training, validation, and evaluation, respectively. During training, class weights were applied such that the less-represented class would have, on average, equal loss as the over-represented class in a completely naive, untrained classifier.

Two-photon calcium imaging

We imaged activity of GCaMP6s-expressing Gad1+ cells in the central brain following P1a optogenetic activation (P1a>Chrimson, Gad1>GCaMP6s; Table S2) using a custom-built two-photon laser scanning microscope. Virgin male flies (3-7 days old) were positioned with the posterior surface of the brain up. Cuticle, air sacks, trachea, and fat were removed to create an imaging window. Saline was heated to 30 degrees centigrade and continuously delivered to the fly head. We recorded both hemispheres simultaneously with a volume rate of 1.4 Hz. The imaging volume size was 200 x 400 x 40 um3 (covering the majority of the central brain) with a pixel resolution of 128 × 256 × 200. For P1a activation, we used a stimulus of 2 seconds ON (at 17 mW/cm2 irradiance), with an inter-stimulus interval drawn from a normal distribution with a mean of 25 seconds, a hard lower limit of 15 seconds, and a hard upper limit of 45 seconds. To process the GCaMP6s signal, we motion-corrected each volume to a template volume using the ANTsPy registration library. Slices within each volume were temporally resampled to correct for different slice timing across planes of the same volume. ROI temporal dynamics and spatial footprints were identified using the Constrained Nonnegative Matrix Factorization (CNMF) algorithm as implemented in CaImAn ([10, 11]. ROI footprints for each fly were warped into a common Gad1 mean brain space to compare spatial distributions across animals. F1 ROIs were identified by fitting a linear model to estimate the optogenetic modulation strength for each ROI compared to a null distribution of activations, which was generated by systematically shifting the opto ON times relative to the true times. F2 ROIs were identified by computing Pearson correlation coefficients and statistical significance scores between all F1 ROIs and all other ROIs around the time of optogenetic activation. ROIs were considered part of the F2 population if they had both negative correlations with respect to an F1 ROI and a statistically significant correlation after Holm-Bonferonni multiple comparisons correction (p<0.05, adjusted).

Closed-loop neural activation

For closed-loop neural activation, stimulus LEDs were turned on at $51.5\mu\text{W}/\text{mm2}$), triggered on the detection of sine song, using fast online sine segmentation (see above). For yoked control experiments, the stimulus of a closed-loop experiment was routed to the second chamber, such that stimulus statistics were identical between experiments but the correlation to male behavior was lost in the yoked control. In each experiment, the two chambers were randomly chosen for closed-loop activation or yoked control.

Patch clamp recordings

Newly eclosed CsChrimson-expressing male flies were collected and raised on food that contained all-trans retinal for 24h before experiments. Flies were anesthetized on ice and mounted on a fly holder using ultraviolet glue. The cell bodies of pIP10 were made accessible by removing part of the cuticle, some trachea, and the perineural sheath covering the posterior brain. During the *in vivo* patch-clamp experiment, the fly dorsal head was continuously perfused with extracellular saline (103mM NaCl, 3mM KCl, 5mM N-Tris (hydroxymethyl)methyl-2-aminoethane-sulfonic acid, 8mM trehalose, 10mM glucose, 26mM NaHCO3, 1mM NaH2PO4, 1.5mM CaCl2 and 4mM MgCl2, 275–280mOsm, pH 7.3) bubbled with 95% O2 and 5% CO2. pIP10 neurons were identified as fluorescent using mVenus and were recorded on either side of the brain, using wavesurfer software (version 0.982,`https://wavesurfer.janelia.org/`) run via Matlab 2018b, and using glass electrodes with tip-resistances between 5–7 MΩ, filled with intracellular saline (140mM potassium aspartate, 10mM HEPES, 5mM EGTA, 4mM MgATP, 0.5mM Na3GTP, 1mM KCl, adjusted to 260–270mOsm, pH 7.3). Negative and positive current pulses of five seconds (with ten seconds between pulses) were injected into pIP10. Recordings were obtained using a MultiClamp 700B amplifier and then digitized with a NiDAQ PCI-6251 (National Instruments) at 10 kHz. Corrections of liquid junction potential correction were not performed.

Immunohistochemistry

Fly dissections were performed on 3-6-day-old adult males in chilled phosphate-buffered saline (PBS). The dissected brains and VNSs were fixed in 2% paraformaldehyde in PBS with 0.1% Triton X-100 (PBT) for 55 minutes at room temperature and then were washed ten times for 5 min each with 0.5% PBT. Following fixation, brains and VNCs were moved to a blocking solution of 5% normal goat serum in 0.1% PBT for 2h at room temperature and then were transferred to a primary antibody solution for incubation for 3d at 4°C. The primary antibody solution contains mouse anti-Bruchpilot (nc82, 1:30, Developmental Studies Hybridoma Bank) and chicken anti-GFP (1:1,000, 1:1000) diluted in the blocking solution. After primary incubation, brains and VNCs were washed ten times for 5 min each with 0.5% PBT before they were moved to a secondary antibody solution for incubation for 4 hr at room temperature and then 24 hr at 4°C. The secondary antibody solution contains Alexa 488-conjugated goat anti-chicken (1:250, Invitrogen) and Alexa 568-conjugated goat anti-mouse (1:250, Invitrogen) diluted in the blocking solution. After ten final washes for 5 min each with 0.5% PBT, brains and VNCs were mounted on slides in Vectashield (Vector Laboratories) for confocal imaging.

Brains and VNCs were imaged using a Leica confocal microscope (TCS SP8) with either a Leica HC PL APO 20x/0.75 CS2 objective or a Leica HC PL APO 63x/1.40 Oil CS2 objective. Maximum intensity projections were generated from stacks of optical sections (taken at 0.69-2um spacing) using Imaris 9.8 (Oxford Instruments).

Pulse type classification

As part of offline song segmentation, detected pulses were classified into two pulse types (Pfast and Pslow) using the methodology described in [7]. Briefly, the pulse classifier takes as input the pulse waveforms returned by the offline song segmenter and assigns pulse type based on the similarity of the pulse waveform to templates for each pulse type.

1 References

[1] Philip Coen, Jan Clemens, Andrew J Weinstein, Diego A Pacheco, Yi Deng, and Mala Murthy. Dynamic sensory cues shape song structure in drosophila. *Nature*, 507(7491):233–237, 2014.

[2] Yun Ding, Joshua L Lillvis, Jessica Cande, Gordon J Berman, Benjamin J Arthur, Xi Long, Min Xu, Barry J Dickson, and David L Stern. Neural evolution of context-dependent fly song. *Current biology*, 29(7):1089–1099, 2019.

[3] Eric D Hoopfer, Yonil Jung, Hidehiko K Inagaki, Gerald M Rubin, and David J Anderson. P1 interneurons promote a persistent internal state that enhances inter-male aggression in drosophila. *Elife*, 4:e11346, 2015.

[4] David Deutsch, Jan Clemens, Stephan Y Thiberge, Georgia Guan, and Mala Murthy. Shared song detector neurons in drosophila male and female brains drive sex-specific behaviors. *Current biology*, 29(19):3200–3215, 2019.

[5] Troy R Shirangi, Allan M Wong, James W Truman, and David L Stern. Doublesex regulates the connectivity of a neural circuit controlling drosophila male courtship song. *Developmental Cell*, 37(6):533–544, 2016.

[6] Yufeng Pan, Carmen C Robinett, and Bruce S Baker. Turning males on: activation of male courtship behavior in drosophila melanogaster. *PloS one*, 6(6):e21144, 2011.

[7] Jan Clemens, Philip Coen, Frederic A Roemschied, Talmo D Pereira, David Mazumder, Diego E Aldarondo, Diego A Pacheco, and Mala Murthy. Discovery of a new song mode in drosophila reveals hidden structure in the sensory and neural drivers of behavior. *Current Biology*, 28(15):2400–2412, 2018.

[8] François Chollet et al. keras, 2015. URL `https://github.com/fchollet/keras`.

[9] James Bergstra, Olivier Breuleux, Frédéric Bastien, Pascal Lamblin, Razvan Pascanu, Guillaume Desjardins, Joseph Turian, David Warde-Farley, and Yoshua Bengio. Theano: a cpu and gpu math expression compiler. In *Proceedings of the Python for scientific computing conference (SciPy)*, volume 4, pages 1–7. Austin, TX, 2010.

[10] Andrea Giovannucci, Johannes Friedrich, Pat Gunn, Jeremie Kalfon, Brandon L Brown, Sue Ann Koay, Jiannis Taxidis, Farzaneh Najafi, Jeffrey L Gauthier, Pengcheng Zhou, et al. Caiman an open source tool for scalable calcium imaging data analysis. *Elife*, 8:e38173, 2019.

[11] Diego A Pacheco, Stephan Y Thiberge, Eftychios Pnevmatikakis, and Mala Murthy. Auditory activity is diverse and widespread throughout the central brain of drosophila. *Nature neuroscience*, 24(1):93–104, 2021.
